# High-pressure treatment enhanced aromatic compound concentrations of melon juice and its mechanism

**DOI:** 10.3389/fnut.2022.1052820

**Published:** 2022-12-01

**Authors:** Xiao Liu, Ruiqi Wang, He Liu, Yubin Wang, Yue Shi, Chao Zhang

**Affiliations:** ^1^Institute of Agri-Food Processing and Nutrition, Beijing Academy of Agriculture and Forestry Sciences, Beijing Key Laboratory of Agricultural Products of Fruits and Vegetables Preservation and Processing, Key Laboratory of Vegetable Postharvest Processing, Ministry of Agriculture and Rural Affairs, Beijing, China; ^2^College of Food Science and Technology, Bohai University, Jinzhou, China

**Keywords:** melon juice, high pressure treatment, GC-MS analysis, β-ionone, surface tension, weight loss rate

## Abstract

**Introduction:**

The flavor deterioration blocks the development of melon juice.

**Methods:**

The effects of ultra-high temperature (UHT) and high pressure (HP) treatments on the aromatic compound concentrations of melon juice and their mechanisms were explored with fresh juice as the control.

**Results:**

A total of 57 volatile compounds were identified by gas chromatography-tandem mass spectrometry analysis. β-ionone was shown to be the major aromatic component of melon juice for the first time. The HP at 200 MPa for 20 min increased the total volatile concentration of melon juice by 1.54 and 3.77 times the control and UHT, respectively. Moreover, the sum concentration of a major aromatic component in the HP treatment was 1.49 and 5.94 times higher than that of the control and UHT, respectively.

**Discussion:**

The HP treatment raised the concentration of volatile and aromatic components of melon juice by reducing their surface tension.

## Introduction

Melons (*Cucumis melo* L.) are favored by people worldwide for their distinctive aroma and sweetness. However, commercialized production of melon juice is hard to realize due to the unsolved obstacle of flavor deterioration (1). Ultra-high temperature (UHT) is the most widely used juice sterilization technology. However, the UHT also leads to serious flavor deterioration. The UHT treatment of sea buckthorn juice resulted in a decrease of 3.48 and 14.60% in total volatiles and esters at 140°C for 2 s and a sharp decrease of 6.90% at 140°C in alcohol contents (2).

Moreover, the UHT also caused an unacceptable cooked off-flavor odor similar to mature pumpkin in melon juice. Dimethyl sulfide, methional, methanethiol, dimethyl trisulfide, dimethyl disulfide, and acetaldehyde have been identified as the off-flavors (3). The formation of volatile sulfur compounds has been inhibited by reducing the pH of melon juice to 2.0 or adding epicatechin (4). However, this method was difficult to use in commercialized production.

High-pressure (HP) technology is a non-thermal process that applies high hydrostatic pressure to the food matrix through a specific liquid transfer medium (5). Compared with the thermal treatment, the HP had a lower impact on the nutrition and flavor of foods due to its better control of the temperature during processing (6, 7). For foods with high moisture content, the temperature increased to ~3°C per 100 MPa. Therefore, HP processing led to better flavor retention for fruit juice. Recent studies showed that the HP treatment (500 MPa for 10 min) of kiwifruit juice maintained the retention rate of characteristic aromas from esters and alcohols (8). Similar results were also demonstrated in the pineapple juice (9). Despite most studies focusing on flavor retention, the effects of HP processing on the concentrations of melon juice flavor and their mechanism were ignored.

This study explored the effect of the UHT and HP treatments on the aromatic compounds of melon juice and their potential mechanism. Specifically, the concentration of volatile components of the UHT and HP treatments was measured by the GC-MS analysis with the fresh juice as the control. The major aromatic compounds were compared to determine the optimal HP parameters. The physical properties of the optimal HP and UHT treatments were compared to discover the potential mechanism.

## Materials and methods

### Experiment design

The UHT and HP treatments were compared with the fresh melon juice as the control. Melon was purchased from Guoxiangsiyi Fruit Supermarket in June 2021 in Haidian District, Beijing. The melon (*C. melo* L. var. Xizhoumi No. 25) was oval and light gray with a shallow net and weighed about 1.2–2.5 kg per fruit. The flesh of the fruits was light orange and crispy, with a soluble solid concentration of 9.5%−13%.

#### Control

The surface of the fruits was washed in an icy sodium hypochlorite solution (100 mg/L) and flushed two times with icy water. The peel and seeds were removed in a sanitary processing workshop. The flesh was cut into cubes and smashed in a Philips juicer for 5 min (HR1861, Philips Ltd., Beijing, China). After quickly removing the top foam, the mixture was sealed in an aluminum foil bag of 200 mL and stored in a refrigerator at −4°C for subsequent sample determination.

#### UHT

The fresh melon juice was sterilized in the ultra-high temperature unit (FT74X-40-44-A, Armfield Ltd.) at room temperature. Then, 1.5 L of juice were poured into the UHT equipment's feeder and heated at 135°C for 15 s. For the subsequent analysis, the sterilized sample was quickly sealed and cooled in an icy bath in a 200-ml aluminum foil bag.

#### HP

The fresh melon juice was processed in the ultra-high pressure unit (BDS200-FL, Stansted Fluid Power Ltd., England) at room temperature. The melon juice sealed in the 200-mL aluminum foil bag was subjected to six kinds of treatments: (1) 200 MPa for 10 min; (2) 200 MPa for 20 min; (3) 400 MPa for 10 min; (4) 400 MPa for 20 min; (5) 600 MPa for 10 min; and (6) 600 MPa for 20 min. They were nominated as HP2-1, HP2-2, HP4-1, HP4-2, HP6-1, and HP6-2, respectively. The holding time did not include the time to increase and release the pressure. After reaching the pressure holding time, the system automatically released the pressure within 10–20 s. The pressured sample was cooled in an icy bath quickly for subsequent analysis.

### Analysis of volatile compounds

The volatile compounds were detected by using a headspace solid-phase microextraction tandem gas chromatography-mass spectrometer (GC-MS) method, as described by Luo et al. (10), with a few modifications. The sample (6.0 g) was transferred into 20-ml headspace glass vials containing 2.0 g of sodium chloride and 10 μl of octanol (30 μg/ml) as an inner standard. The sample was stirred at 100 rpm, and its volatile compounds in the headspace were extracted and absorbed by an SPME fiber (57329-U PDMS/DVB/CAR, Sigma-Aldrich Company, USA) at 50°C for 30 min. After being absorbed, the absorbed compounds were thermally desorbed at 250°C for 3 min in a splitless mode by a GC-MS system (6890N/5977B, Agilent Technologies Company, USA). Volatile compounds were separated on a DB-5MS elastic capillary column (30 m × 0.25 mm × 0.25 μm; Agilent Technologies, USA). Helium was used as a carrier gas with a constant flow rate of 1.0 ml/min. The initial temperature in the oven was set at 35°C for 5 min and increased at a rate of 4°C/min to 150°C, held for 3 min, and increased at a rate of 8°C/min to 190°C, again held for 1 min, and ramped to 250°C at 30°C/min, and held at 250°C for an additional 5 min. The full scan mode was adopted to collect signals at a scan speed of 1,562 u/s. The mass detector was operated in electron impact mode (70 eV). The ion source temperature was 230°C, the transmission line temperature was 250°C, and the quadrupole temperature was 150°C. The detected volatile compounds were identified by comparing the mass spectra with those in mass spectral libraries (NIST17). An MS match index of ≥80% was listed and verified manually, point by point. The concentration of each aromatic compound was calculated based on the peak areas of 1-octanol, an internal standard with a known concentration (Equation 1):


(1) $$\begin{array}{l} m_{x}=\frac{C_{i}\times V_{i}\times A_{x}}{m_{s}\times A_{i}}\times 1,000 \end{array}$$

where *Ci* refers to the mass concentration of the standard internal compound and the unit was μg/ml; *Vi* refers to the additional amount of the internal standard in the sample, 10 μl; *ms* refers to the sample mass of 6 g; *Ax* and *Ai* refer to the peak areas of the target compound and the standard internal compound, respectively. *mx* refers to the concentration of the target compound, expressed in μg/kg fresh weight (FW).

### Calculation of odor activity values

The odor activity values (OAV) were the ratio of the concentration to their corresponding odor threshold in water (11) and were calculated according to Equation (2). Normally, compounds with OAVs of no < 1.0 were potential flavoring agents:


(2) $$\begin{array}{l} OAV_{i}=\frac{C_{i}}{O_{i}} \end{array}$$

where *Ci* is the concentration of the compound, and *Oi* is the odor threshold of the compound.

### Surface tension analysis

The juice sample of 50 ml was put into the glass container of the surface tension tester (K100C-MK2, KRUSS, Germany). The surface tension was tested with the platinum tablet plate at 25°C. The instrument was calibrated with water. The testing parameters were set as follows: the measurement speed was 10 mm/min, the immersion depth was 2.00 mm, the maximum measurement time was 60 s, and the deviation value was 0.1 mN/m. The result was the average of five measured values with a stable measurement of less than the deviation value. The platinum tablet plate was thoroughly cleaned and flame-dried before each measurement.

### Thermogravimetric analysis

The thermogravimetric differential thermal synchronous analyzer (TGA/DSC 1, Mettler Toledo, Switzerland) was preheated for half an hour. The crucibles were heated at 500°C before the test. The heating chamber was preheated to 60°C in advance. The 25-μL sample was then loaded into the crucibles and placed isothermally in the chamber at 60°C for 20 min, with distilled water serving as the control. The weight loss rate was the mass loss caused by sample evaporation, which was determined in an area with constant temperature and varied linearly with time (12). The curve of the weight loss rate in **Figure 6A** was calculated after the original quality data was normalized. The curve in **Figure 6B** is named *Y*′, which indicates the normalized data of melon juice samples minus the water sample.

### Statistical analysis

All the measurements were repeated three times. A one-way analysis of variance was conducted on different groups using SPSS Statistics 26.0. The results were shown as mean ± standard deviation at a significance level of a *P*-value of < 0.05. The graphs were all plotted using Origin 2021. The profile of the mechanism was prepared with PowerPoint.

## Results and discussion

### Identification of volatile compounds in melon juice

A total of 57 volatile components were detected in control, UHT treatment, and HP treatment, including 20 esters, 15 alcohols, 14 aldehydes, and eight ketones (Table 1). The volatile component number of the control, UHT, HP2-1, HP2-2, HP4-1, HP4-2, HP6-1, and HP6-2, was 16, 37, 19, 20, 25, 29, 25, and 26, respectively. The UHT included more volatile components than the other treatments. The composition of volatile compounds in the control and HP groups was similar, such as ethyl acetate and nonanal. There were clear differences between the control and UHT treatment. In addition, with the increase of HP parameters between HP groups, the same components as those in the UHT group appear in the HP4-1, HP4-2, HP6-1, and HP6-2 groups, such as (Z)-3-hexenyl acetate and decyl aldehyde. These volatile components were combined to form the final flavor of melon juice.

**Table 1:** Identification and concentrations of volatile components of melon juice.

**ID**	**Volatile components^1^**	**CAS**	**Molecular formula**	**Control (μg/kg)^2^**	**UHT (μg/kg)^2^**	**HP2-1 (μg/kg)^2^**	**HP2-2 (μg/kg)^2^**	**HP4-1 (μg/kg)^2^**	**HP4-2 (μg/kg)^2^**	**HP6-1 (μg/kg)^2^**	**HP6-2 (μg/kg)^2^**
**Ester**											
1	Diisobutyl phthalate	84-69-5	C_16_H_22_O_4_	–	–	–	–	–	0.42 ± 0.02	–	–
2	Butyl-octyl phthalate	84-78-6	C_20_H_30_O_4_	8.50 ± 2.12	0.43 ± 0.01	–	–	–	–		
3	2-Methyl-1-butyl acetate	624-41-9	C_7_H_14_O_2_	–	0.84 ± 0.02	8.19 ± 0.12	9.76 ± 0.50	9.22 ± 0.32	7.08 ± 1.20	3.45 ± 0.24	3.11 ± 0.32
4	Isoamyl acetate	123-92-2	C_7_H_14_O_2_	–	–	–	–	–	0.73 ± 0.11	–	–
5	2,2,4-Trimethyl-1,3-pentanediol diisobutyrate	6846-50-0	C_16_H_30_O_4_	–	0.95 ± 0.23	–	–	–	–	–	–
6	2,4-Pentanediol,2,4-diacetate	7371-86-0	C_9_H_16_O_4_	–	0.62 ± 0.12	–	–	0.75 ± 0.04	–	–	–
4	1-(Benzoyloxy)-2,5-pyrrolidinedione	23405-15-4	C_11_H_9_NO_4_	–	0.61 ± 0.11	–	2.31 ± 0.02	1.44 ±0.11	–	–	–
8	2-Methylacetic acid-2-alkenyl ester	33425-30-8	C_5_H_10_O·C_2_H_4_O_2_	–	–	–	–	0.52 ± 0.01	0.40 ± 0.03	–	–
9	(Z)-3-Hexenyl acetate	3681-71-8	C_8_H_14_O_2_	–	1.97 ± 0.22	–	–	–	–	0.95 ± 0.01	0.88 ± 0.02
10	2-Ethylhexyl acetate	103-09-3	C_10_H_20_O_2_	–		–	–	–	–	–	0.66 ± 0.05
11	Phenethyl acetate	103-45-7	C_10_H_12_O_2_	–	1.13 ± 0.01	–	–	–	–	–	–
12	Butyl acetate	123-86-4	C_6_H_12_O_2_	1.44 ± 0.25	0.29 ± 0.06	1.75 ± 0.25	2.40 ± 0.26	2.45 ± 0.18	2.47 ± 0.15	1.32 ± 0.13	1.25 ± 0.01
13	(Z)-non-3-enyl ester acetic acid	13049-88-2	C_11_H_20_O_2_	–	0.32 ± 0.03	–	–	–	–	–	–
14	Benzyl acetate	140-11-4	C_9_H_10_O_2_	–	31.09 ± 2.23	–	–	4.55 ± 0.54	3.71 ± 0.25	0.86 ± 0.11	1.08 ± 0.32
15	2,4-Dimethylbenzoate	55000-43-6	C_18_H_20_O_2_	–	1.82 ± 0.02	–	–	–	–	–	–
16	Ethyl acetate	141-78-6	C_4_H_8_O_2_	21.06 ± 2.24	2.38 ± 0.21	18.68 ± 2.12	21.59 ± 2.42	22.98 ± 2.54	16.59 ± 2.01	10.59 ± 1.58	5.54 ± 1.01
17	Isobutyl acetate	110-19-0	C_6_H_12_O_2_	8.14 ± 1.52	1.39 ± 0.23	9.17 ± 1.15	12.25 ± 1.65	10.90 ± 1.12	9.91 ± 1.14	5.33 ± 1.25	4.74 ± 0.52
18	Isopulegol acetate	57576-09-7	C_12_H_20_O_2_	–	–	–	–	–	0.44 ± 0.02	–	–
19	n-Propyl acetate	109-60-4	C_5_H_10_O_2_	–	–	–	–	–	–	–	0.54 ± 0.12
20	Propanoic acid, 2-methyl-, 3-hydroxy-2,2,4-trimethylpentyl ester	77-68-9	C_12_H_24_O_3_	–	0.37 ± 0.03	–	–	–	–	–	–
**Alcohol**											
1	(Z)-6-nonen-1-ol	35854-86-5	C_9_H_18_O	–	3.05 ± 0.52	–	–	–	–	0.91 ± 0.23	–
2	1-Hexanol	111-27-3	C_6_H_14_O	–	–	–	–	–	–	0.36 ± 0.15	–
3	2-Ethylhexanol	104-76-7	C_8_H_18_O	8.71 ± 2.23	2.61 ± 0.56	9.12 ± 2.52	13.55 ± 2.65	7.92 ± 1.25	8.30 ±1.34	4.38 ± 0.98	4.36 ± 1.25
4	1-Nonanol	143-08-8	C_9_H_20_O	24.49 ± 3.65	9.67 ± 2.54	23.88 ± 5.65	25.14 ± 4.25	1.68 ± 0.35	1.73 ± 0.52	4.17 ± 0.87	
5	1-Octen-3-ol	3391-86-4	C_8_H_16_O	–	–	–	–	0.41 ± 0.02	0.56 ± 0.08	0.25 ± 0.03	0.30 ± 0.01
6	2,2,4-Trimethyl-1,3-pentanediol diisobutyrate	6846-50-0	C_16_H_30_O_4_	–	0.95 ± 0.05	–	–	–	–	–	–
7	2-Ethylhex-2-enol	50639-00-4	C_8_H_16_O	–	–	–	–	–	0.45 ± 0.02	–	–
8	2-Nonen-1-ol	22104-79-6	C_9_H_18_O	2.31 ± 0.25	–	1.81 ± 0.12	2.52 ± 0.23	–	–	–	–
9	(E,Z)-3,6-Nonadien-1-ol	56805-23-3	C_9_H_16_O	32.84 ± 5.65	13.54 ± 2.56	32.60 ± 4.25	51.68 ± 5.89	16.26 ± 2.25	15.82 ± 1.12	7.22 ± 2.23	5.80 ± 1.13
10	(E)-3-Hepten-1-ol	2108-05-06	C_7_H_14_O	–	–	–	1.24 ± 0.23	–	–	–	–
11	(Z)-3-Nonen-1-ol	10340-23-5	C_9_H_18_O	127.55 ± 12.25	33.26 ± 8.23	120.96 ± 12.25	183.21 ± 16.25	46.21 ± 4.32	40.75 ± 6.25	15.02 ± 3.25	12.96 ± 3.52
12	1-Methyl-4-(1-methylethenyl)-Cyclohexanol	138-87-4	C_10_H_18_O	–	–	–	–	0.43 ± 0.03	–	–	–
13	3,5-Dimethylcyclohexanol	5441-52-1	C_8_H_16_O	–	–	–	–	–	0.37 ± 0.05	–	–
14	1,8-Oxido-p-menthane(Cineole)	470-82-6	C_10_H_18_O	–	0.42 ± 0.04	–	–	–	–	–	–
15	2-Phenylethanol	60-12-8	C_8_H_10_O	–	1.01 ± 0.12	–	–	–	–	–	–
**Aldehyde**											
1	2,6,6-Trimethyl-1-Cyclohexene-1-acetaldehyde	472-66-2	C_11_H_18_O	–	1.27 ± 0.23	1.67 ± 0.21	2.55 ± 0.25	2.74 ± 0.35	2.98 ± 0.36	1.46 ± 0.15	1.62 ± 0.14
2	2,6,6-Trimethyl-1-cyclohexene-1-carboxaldehyde (β-Cyclocitral)	432-25-7	C_10_H_16_O	–	2.54 ± 0.53	–	–	–	–	–	–
3	2,4-Decadienal	25152-84-5	C_10_H_16_O	–	0.39 ± 0.12	–	–	0.45 ± 0.08	–	0.25 ± 0.04	0.29 ± 0.02
4	(E)-2-Heptenal	18829-55-5	C_7_H_12_O	–	–	10.36 ± 3.59	18.69 ± 2.85	–	–	–	–
5	(E)-2-Nonenal	18829-56-6	C_9_H_16_O	–	–	–	–	–	13.01 ± 1.25	2.26 ± 0.25	1.64 ± 0.52
6	(E)-6-Nonenal	2277-20-5	C_9_H_16_O	11.70 ± 1.23	–	–	–	–	–	–	–
7	(Z)-7-Tetradecenal	65128-96-3	C_14_H_26_O	–	3.97 ± 0.23	–	–	–	–	–	–
8	Phenylacetaldehyde	122-78-1	C_8_H_8_O	–	2.76 ± 0.27	1.62 ± 0.12	–	–	1.00 ± 0.26	–	0.69 ± 0.02
9	Decyl aldehyde	112-31-2	C_10_H_20_O	–	0.52 ± 0.15	–	–	–	0.80 ± 0.02	–	1.05 ± 0.01
10	Heptaldehyde	111-71-7	C_7_H_14_O	–	–	–	–	1.43 ± 0.55	2.12 ± 0.25	1.12 ± 0.23	2.81 ± 0.01
11	Hexanal	66-25-1	C_6_H_12_O	0.94 ± 0.23	–	–	–	–	–	–	–
12	Nonanal	124-19-6	C_9_H_18_O	20.95 ± 2.56	4.26 ± 0.87	27.10 ± 2.25	42.18 ± 5.58	8.52 ± 1.23	7.21 ± 2.21	1.79 ± 0.05	2.19 ± 0.04
13	Octanal	124-13-0	C_8_H_16_O	60.85 ± 9.58	–	68.69 ± 14.22	96.87 ±13.13	76.81 ± 11.02	71.80 ± 5.89	0.79 ± 0.01	0.65 ± 0.01
14	Acetal	105-57-7	C_6_H_14_O_2_	7.50 ± 2.13	2.76 ± 0.12	8.30 ± 1.23	10.59 ± 2.45	8.81 ± 1.23	9.76 ± 2.96	4.24 ± 0.12	3.33 ± 0.02
**Ketone**											
1	2,6-Bis(1,1-dimethylethyl)-4-hydroxy-4-methyl-2,5-Cyclohexadien-1-one	10396-80-2	C_15_H_24_O_2_	6.65 ± 1.12	–	–	15.12 ± 2.23	12.53 ± 4.02	12.39 ± 1.23	5.44 ± 1.01	4.08 ± 0.25
2	Octahydro-1,1,8a-trimethyl-(E)-2,6-Naphthalenedione	57289-17-5	-	–	1.30 ± 0.05	0.76 ± 0.05	1.08 ± 0.54	–	–	–	–
3	4-(2,2,6-trimethyl-7-oxabicyclo[4.1.0]hept-1-yl)-3-Buten-2-one	23267-57-4	C_13_H_20_O_2_	–	–	–	–	0.43 ± 0.25	0.60 ± 0.02	0.29 ± 0.01	0.33 ± 0.02
4	4-Hydroxy-3-methylacetophenone	876-02-8	C_9_H_10_O_2_	–	0.32 ± 0.02	–	–	–	–	–	–
5	6,10-Dimethylundeca-5,9-dien-2-one	3796-70-1	C_13_H_22_O	–	8.74 ± 1.12	6.48 ± 1.12	15.45 ± 2.89	45.62 ± 4.25	50.84 ± 2.02	25.81 ± 0.03	29.04 ± 1.25
6	6-Methyl-5-hepten-2-one	110-93-0	C_8_H_14_O	–	0.75 ± 0.23	–	–	2.20 ± 0.01	2.68 ± 0.02	1.36 ± 0.24	1.54 ± 0.54
7	1,1,3-Trimethyl-3-cyclohexene-5-one	78-59-1	C_9_H_14_O	–	–	0.63 ± 0.04	–	–	–	–	–
8	(E)-4-(2,6,6-Trimethyl-1-cyclohexen-1-yl)-3-buten-2-one (β-Ionone)	79-77-6	C_13_H_20_O	2.48 ± 1.02	2.83 ± 0.25	3.27 ± 0.42	4.12 ± 1.54	4.44 ± 1.12	3.71 ± 0.25	1.82 ± 0.56	2.08 ± 0.01
	**All ester**			**39.14** **±6.13**	**44.20** **±3.53**	**37.79** **±3.64**	**48.31** **±4.85**	**52.81** **±4.84**	**41.74** **±4.91**	**22.51** **±2.32**	**17.78** **±2.37**
	**All alcohol**			**195.89** **±30.01**	**64.51** **±14.62**	**188.36** **±24.79**	**277.34** **±36.63**	**72.89** **±8.22**	**67.98** **±9.38**	**32.31** **±7.74**	**23.42** **±5.91**
	**All aldehyde**			**101.94** **±13.73**	**18.45** **±2.52**	**117.72** **±21.62**	**170.87** **±24.26**	**98.76** **±14.46**	**108.66** **±13.30**	**11.90** **±0.85**	**14.25** **±0.79**
	**All ketone**			**9.13** **±2.14**	**13.95** **±1.67**	**11.14** **±1.63**	**35.76** **±7.20**	**64.21** **±9.65**	**70.22** **±2.54**	**34.72** **±1.85**	**37.07** **±2.07**
	**Total**			**346.09** **±52.01**	**141.11** **±22.34**	**355.01** **±51.68**	**532.27** **±72.94**	**288.67** **±37.17**	**288.59** **±30.13**	**101.44** **±12.76**	**92.51** **±11.14**

^1^Volatile components detected by the GC-MS compared with the standard mass spectrum in the NIST 17 library.

^2^Each value is the mean of triplicate biological samples.

“–” is not detected.

The total volatile concentration of melon juice was highest (532.27 μg/kg) in the HP2-2 group, which was 1.54 and 3.77 times that of the control and UHT, respectively. The HP2-2 increased the concentration of total volatile components in melon juice, while the UHT treatment significantly reduced it.

β-Ionone was detected in each melon juice for the first time. β-Ionone was produced by the cleavage at the C9 and C10 keys from the β-Carotene metabolic pathways (13), which was a common aromatic volatile compound that existed in a variety of fruits, including raspberry juice (14) and apple juice (15), but had not been reported in melons before. The concentration of β-ionone was highest (4.12 μg/kg) in the HP2-2, which was 1.66 and 1.45 times that of the control and the UHT treatment, respectively.

### Screen of major aromatic components and flavor difference analysis

The odor activity values (OAV) reasonably assess aroma effectiveness based on the balance between food substrate and air (16). The OAV was the ratio of aromatic component concentration to the aroma threshold value. When the OAV was >1.0, the aromatic component contributed to its aroma. The greater the OAV, the greater the aromatic component's contribution to the overall aroma. A total of eight major aromatic components were screened from 57 volatile components based on the OAV calculation (Table 2). The major aromatic components included 2-methyl-1-butyl acetate and ethyl acetate. (E, Z)-3,6-nonadien-1-ol, (Z)-3-nonen-1-ol, acetal, nonanal, octanal, and β-ionone.

**Table 2:** Threshold and odor activity values of aromatic compounds of melon juice.

**ID**	**Aromatic compounds**	**Threshold (μg/kg)**	**OAV** ^**1**^							
			**Control**	**UHT**	**HP2-1**	**HP2-2**	**HP4-1**	**HP4-2**	**HP6-1**	**HP6-2**
1	2-Methyl-1-butyl acetate	8	1.06	0.48	1.02	1.22	1.15	0.89	0.43	0.39
2	Ethyl acetate	5	4.21	0.48	3.74	4.32	4.60	3.32	2.12	1.11
3	(E,Z)-3,6-Nonadien-1-ol	3	10.95	4.51	10.87	17.23	5.42	5.27	2.41	1.93
4	(Z)-3-Nonen-1-ol	1	127.55	33.26	120.96	183.21	46.21	40.75	15.02	12.96
5	Acetal	4.9	1.53	–	1.69	2.16	1.80	1.99	0.87	0.68
6	Nonanal	1.1	19.04	3.87	13.26	38.34	7.74	6.56	1.63	1.99
7	Octanal	0.587	103.66	–	117.01	165.02	130.85	122.31	1.34	1.10
8	β-Ionone	0.007	353.98	404.70	467.51	587.87	491.79	530.64	259.38	296.50
	**Total**		**621.99**	**447.30**	**736.06**	**999.37**	**689.55**	**711.72**	**283.20**	**316.66**

^a^Each value is the mean of triplicate biological samples.

“–”is not detected.

Ester was a key component in the flavor of melon juice (17). 2-Methyl-1-butyl acetate was a branched chain ester showing apple aroma and fragrance (18). Ethyl acetate was not only the major aromatic component of melon but also the origin of most fruit aromas (13, 19). (E, Z)-3,6-nonadien-1-ol showed a strong aroma of cucumber (20). (Z)-3-nonen-1-ol was the major aromatic component of melon, showing a grassy aroma (21). The C6–C9 aldehydes were the key component providing the main flavor for melon juice (22). Acetal and nonanal showed an orange aroma and grassy aroma (14). Octanal had an immature orange aroma (19, 23).

**Figure 1:**
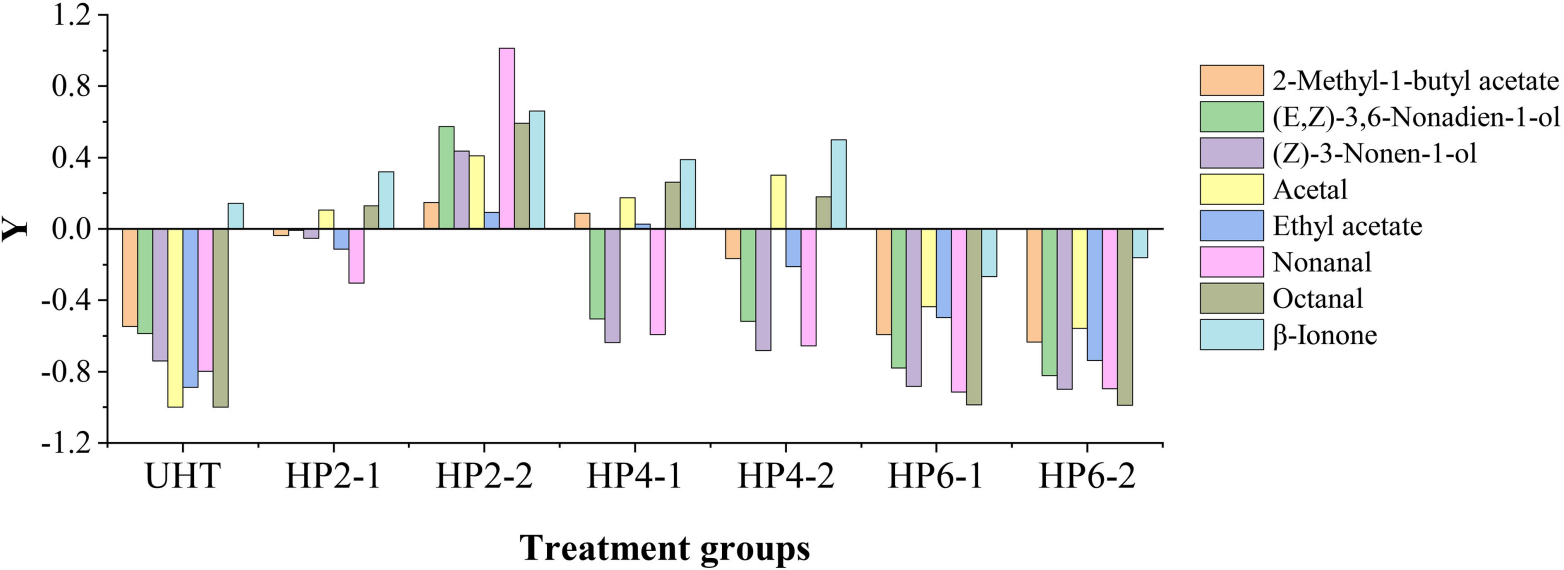
Changes of aromatic components in UHT and HP treatments with the control as a reference. *Y* = *Qi*/*Qc* – 1, *Qi* was the OAV values of aromatic components of the different treatments; *Qc* was the OAV values of aromatic components of the control.

The total OAV of the HP2-2 was 1.61 and 2.23 times that of the control and UHT, respectively (Table 2). The HP2-2 significantly enhanced the flavor of the aromatic components of the melon juice. Moreover, the effect of treatments on the OAV value of aromatic components is shown in Figure 1. *Y* value represents the times of the OAV value of aromatic components to that of the control. The positive value meant an increase in the components, while the negative value meant a decrease in them. The *Y* values of the major aromatic components of all treatments were negative, except for that of the HP2-2. The UHT, HP6-1, and HP6-2 reduced the *Y* value significantly. The *Y* value of all aromatic components in the HP2-2 was positive. Therefore, the HP2-2 significantly enhanced the content of the major aromatic components of the melon juice. However, the *Y* values of the HP6-1 and HP6-2 were similar to that of the UHT. This phenomenon indicated that the excess pressure of the HP treatment led to the deterioration of the aroma. Similar results were reported in the mango juice (24). Therefore, the optimum parameters of the HP processing were 200 MPa for 20 min for the melon juice.

**Figure 2:**
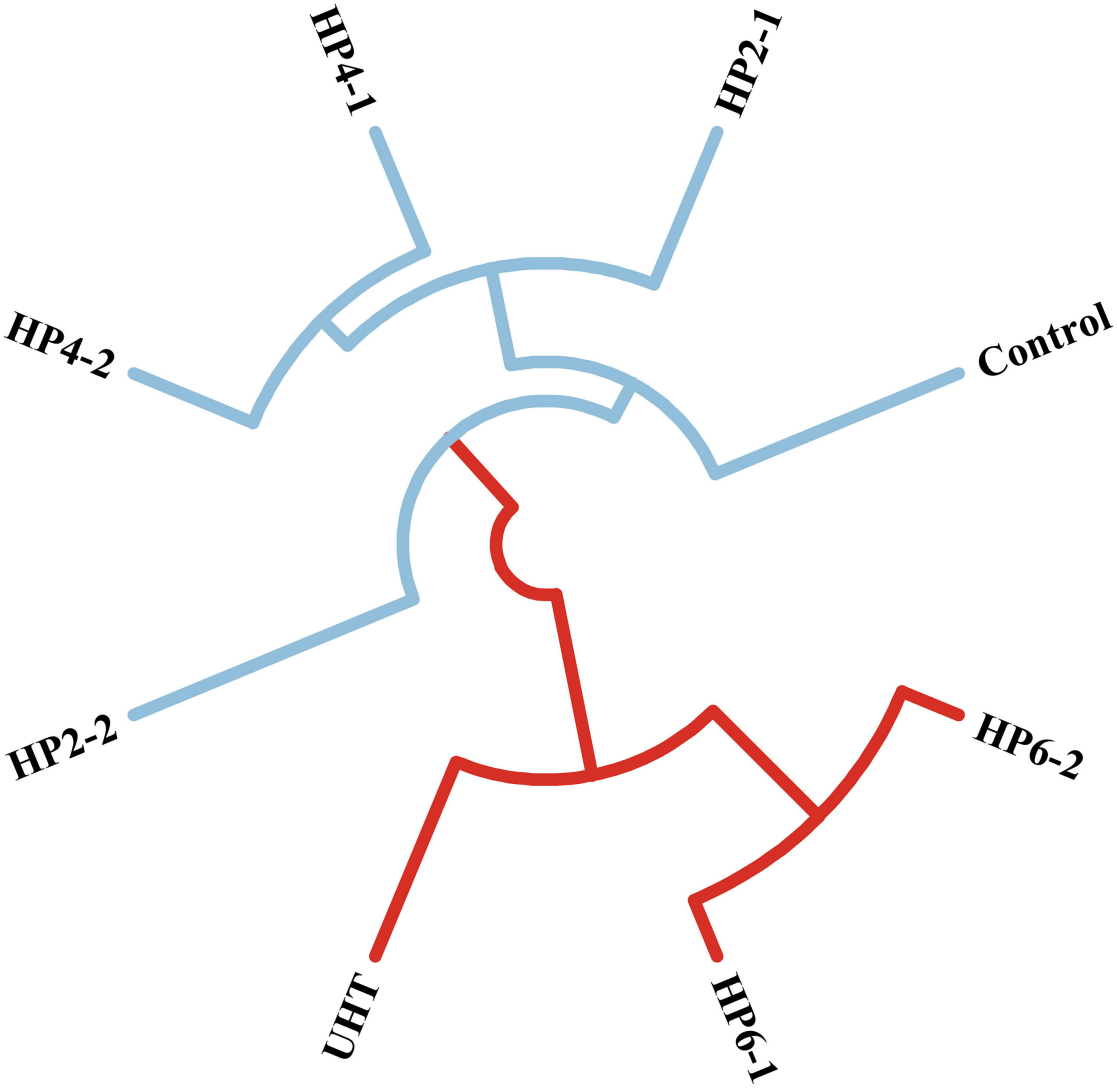
Cluster analysis of the eight major aromatic compounds in the control, UHT and HP.

The cluster analysis was used to find the flavor difference between treatments (Figure 2). The composition of aromatic components from the HP2-1, HP2-2, HP4-1, and HP4-2 was similar to those of the control, with that of the HP2-2 being the most similar. This phenomenon was consistent with the result of the OAV evaluation. Similar results were also reported in cloudy pomegranate juice (25), strawberry juice (26), and mulberry juice (27). The UHT was similar to the HP6-1 and HP6-2. The reason for this was the significant change in the flavor of melon juice due to the temperature change caused by the excessive pressure.

Therefore, the HP2-2 enhanced the aromatic components of melon juice.

### Effects of treatments on the concentration of major aromatic components of melon juice

**Figure 3:**
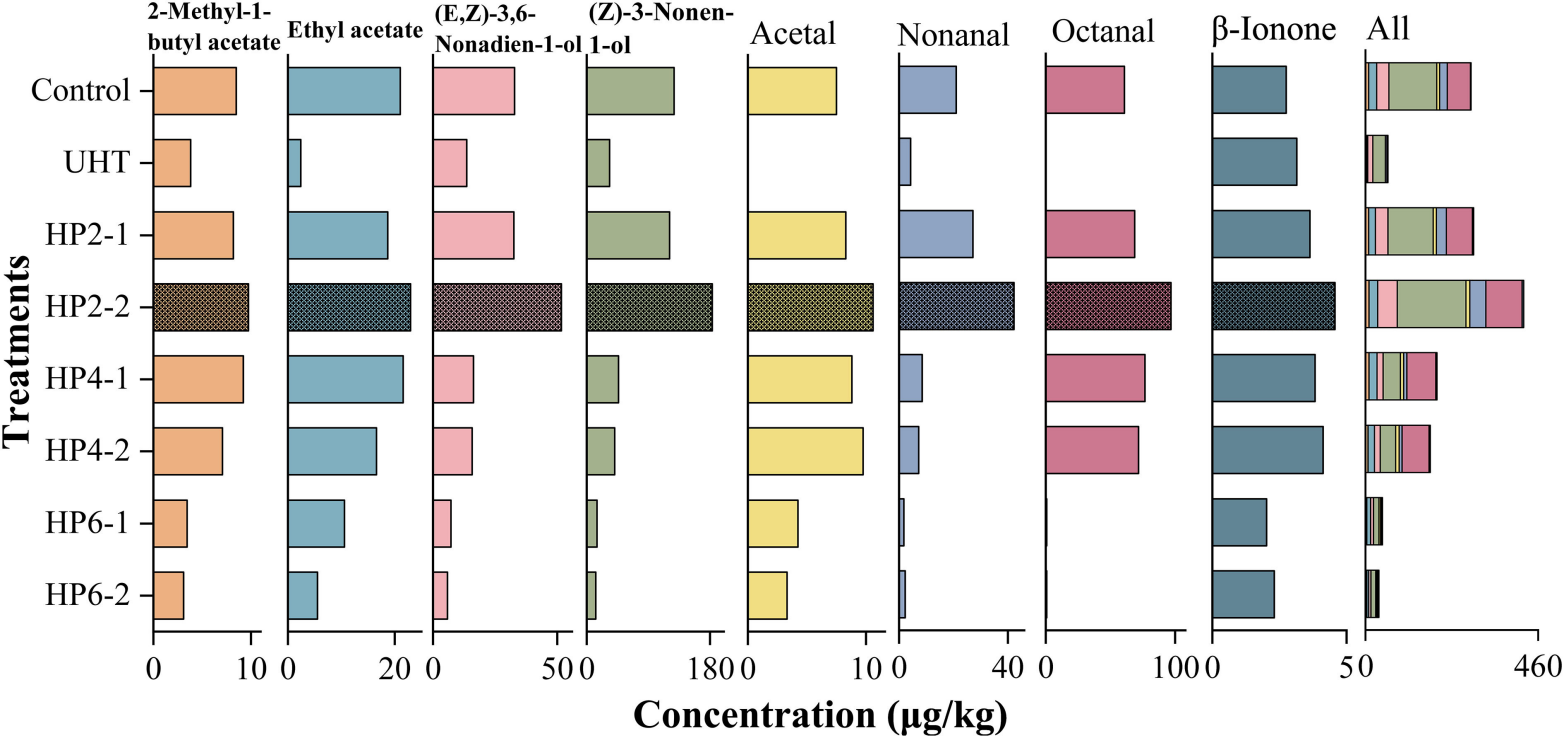
The concentration of eight major aromatic compounds of melon juice. Each value is the mean of triplicate biological samples.

Figure 3 shows the effect of treatments on the concentration of major aromatic components of melon juice. The UHT significantly reduced the concentration of eight aromatic components in all treatments. The total concentration of eight aromatic components in the HP2-2 group was highest, which was 1.49 and 6.99 times that of the control and UHT, respectively.

The concentrations of 2-methyl-1-butyl acetate and ethyl acetate were significantly decreased by the UHT. This phenomenon was probably because the thermal treatment of the UHT was more intensive than the non-thermal treatment of the HP (28).

The HP2-2 increased the concentration of (E, Z)-3,6-nonadien-1-ol and (Z)-3-nonen-1-ol in each treatment. This phenomenon was possible because the proper pressure activated the activity of certain glycosidases and released glycoside-bound alcohols in fruit juices (8).

The UHT reduced acetal, nonanal, and octanal concentrations to a very low level. The high temperature had the greatest effect on aldehydes. Consistent with our results, the aldehydes showed the highest thermal sensitivity and the lowest thermal stability in melon juice, so the high temperature significantly reduced the odor intensity (19).

β-Ionone was the only flavor compound whose concentration was increased by the UHT. The first-order kinetics of β-carotene degradation products can only be produced after long-term exposure to high temperatures, which also promotes the formation of β-ionone (29). The HP2-2 increased the concentration of β-ionone to the maximum, 1.66 and 1.45 times the control and UHT, respectively.

Interestingly, the HP6-1 and HP6-2 reduced the concentrations of major aromatic components, and some of them were reduced to an undetectable level, which resulted from the fact that the excessive pressure destroyed the structure of aromatic compounds. A similar phenomenon was also reported: the HP treatment of 200–400 MPa maintained the volatile components of pumpkin, while the excessive pressure reduced them (30).

### Possible mechanisms of the HP treatment

The aromatic components of melon juice were mostly C6 and C9 aldehydes and their corresponding alcohols, which were mainly products of the fatty acid metabolism catalyzed by the related enzymes (22). Remarkably, the HP treatment would inactivate the enzymes and terminate the synthesis of aromatic components (31–33). However, the concentration of aromatic components in the HP2-2 was significantly higher than that of the control, as indicated by the GC-MS results in our study. These phenomena proved that the enhancement of the aromatic components of the HP2-2 did not result from the catalysis of the related enzymes in the fatty acid metabolism. Research showed that the HP treatment enhanced van der Waals interaction by reducing the C–C bond lengths, which led to molecular aggregation (34, 35). The molecular aggregation reduced the surface tension of the liquid (36, 37), thus enhancing the volatilization (38, 39).

**Figure 4:**
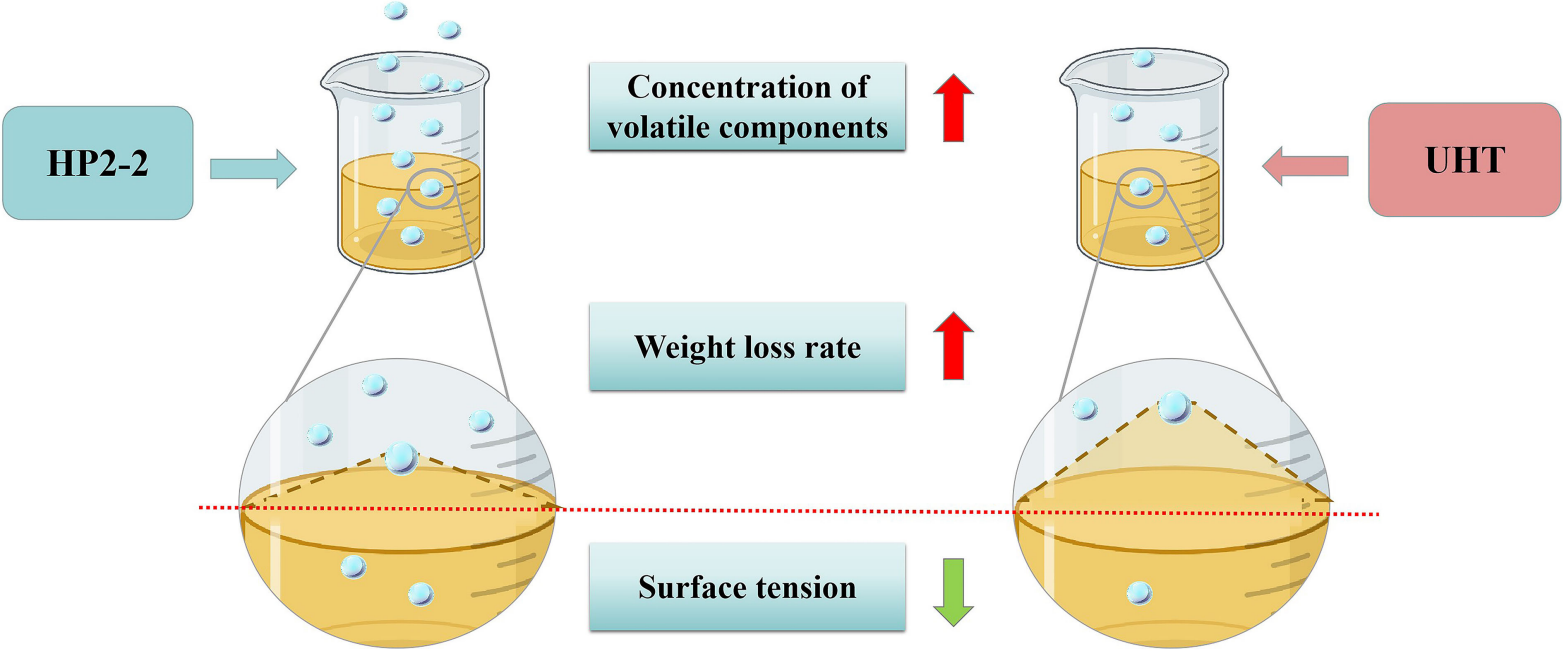
The mechanism profile of the UHT and HP2-2 on aromatic components of melon juice. The blue ball refers to the aromatic components in melon juice.

Consequently, the surface tension was an important factor affecting the aromatic components of the melon juice. The surface tension reduction would raise the liquid volatilization of the juice. The liquid volatilization could be expressed through weight loss. Hence, the surface tension and weight loss of melon juice were evaluated (Figure 4).

**Figure 5:**
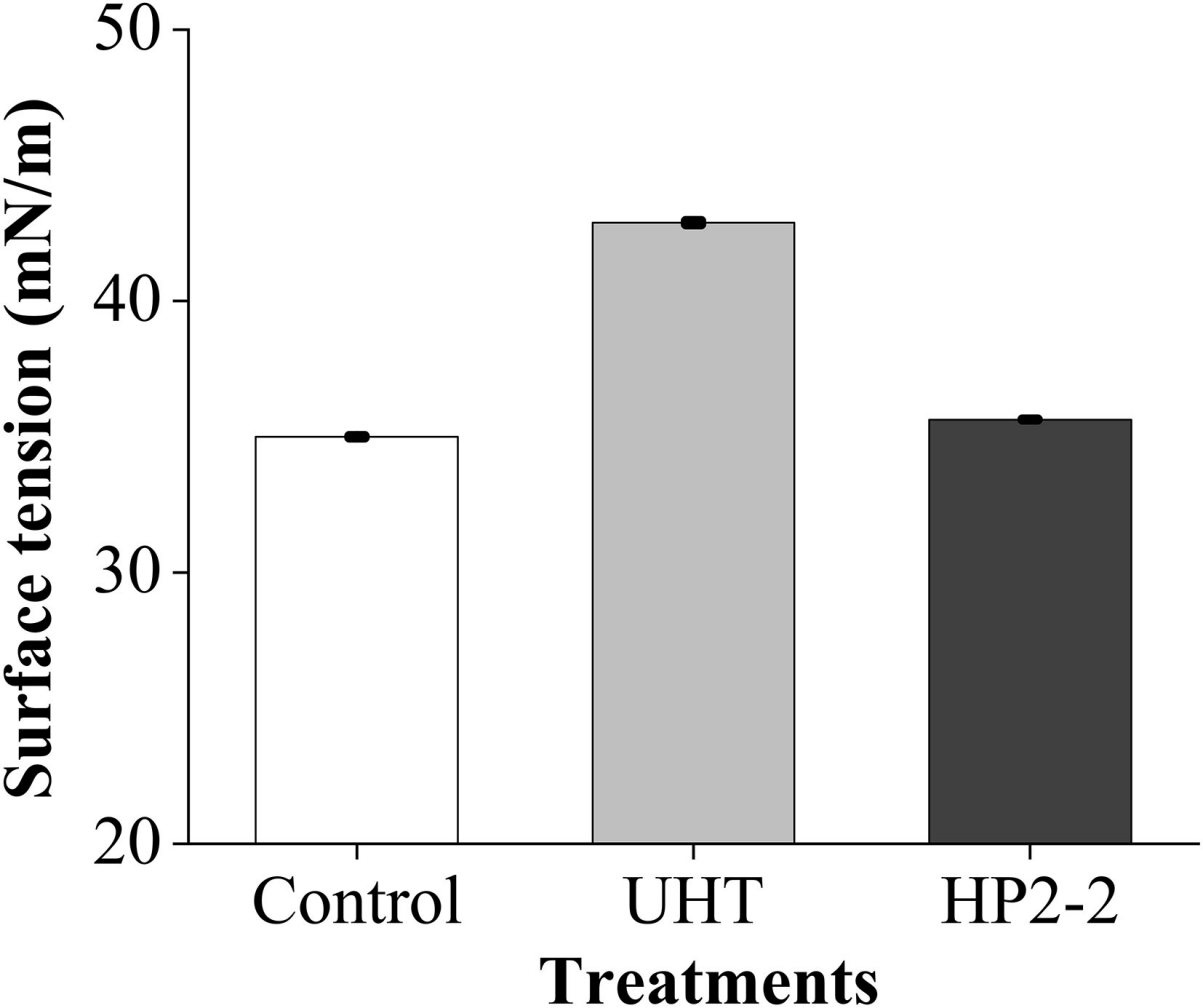
Surface tension of the melon juice. Each value is the mean of triplicate biological samples. The different letters in control, UHT, HP2-2 indicate significant differences (*P* < 0.05).

The surface tension of the UHT treatment was the highest, which was 1.23 times and 1.20 times that of the control and HP2-2, respectively (Figure 5). The surface tension of the HP2-2 was similar to that of the control. Research showed that the reduced surface tension would promote evaporation and recoiling properties (40). The reduction of surface tension will make the aromatic components more volatile. The HP2-2 reduced the surface tension between aromatic components and the water matrix, thereby enhancing the volatilization of aromatic components. Similar results also proved that a pressure higher than 150 MPa would reduce the molecular force between aromatic components and the water matrix (41).

**Figure 6:**
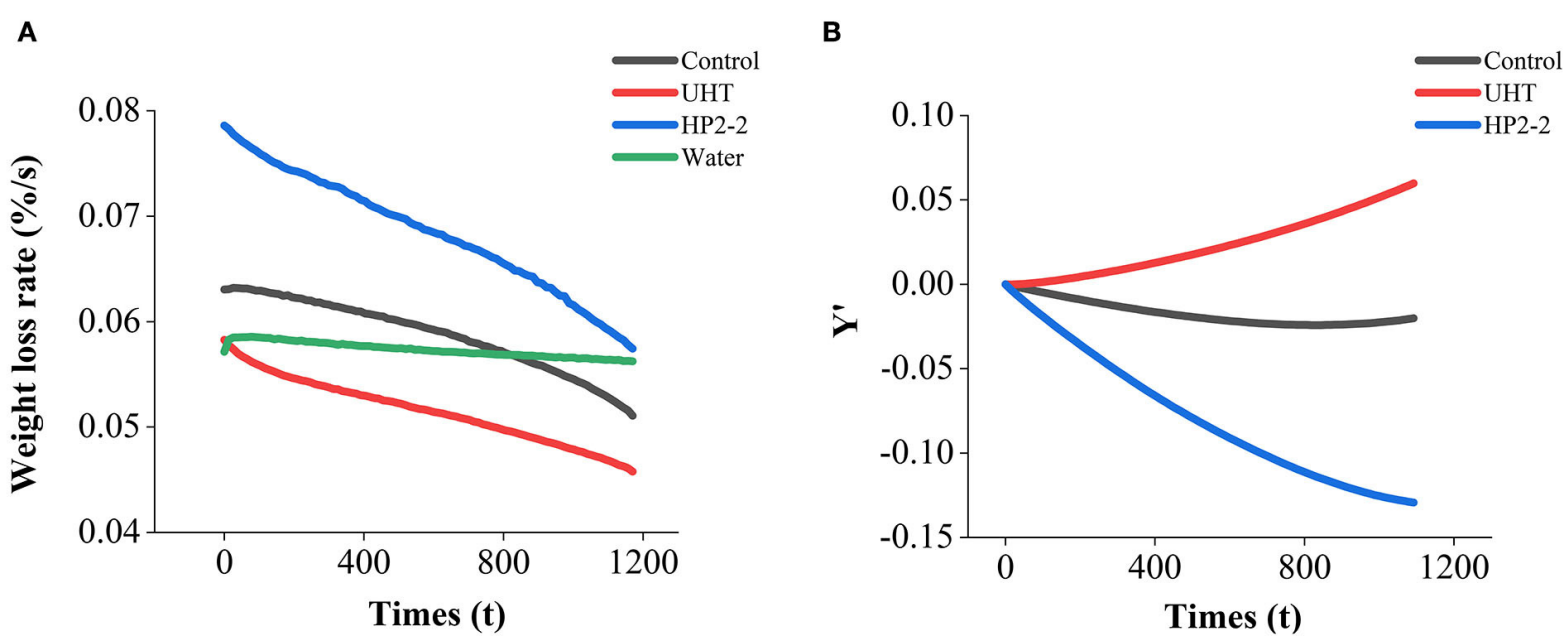
**(A)** Weight loss rate of melon juice and water. **(B)** Weight loss rate of melon juice samples minus water weight loss rate respectively. Each value is the mean of triplicate biological samples. *Y*′ is the quality of melon juice samples minus water during evaporation after normalization.

In Figure 6A, the weight loss rate of the melon juice decreased, while that of the water was held constant. The decrease resulted from the evaporation of moisture in the juice and the increase in melon juice concentration. On the one hand, the energy required to evaporate the same amount of water per unit of time was increased (42). Since the temperature of the TGA/DSC analysis was isothermal at 60°C and the energy provided was constant, the evaporation flux was reduced, and the weight loss rate was also reduced accordingly.

Meanwhile, the increase in melon juice concentration led to an increase in viscosity, which reduced the transfer coefficient in the liquid phase (43). Increasing the solution's viscosity improved the resistance to mass transfer in the liquid phase (44). Consequently, the melon juice showed a polarization effect and induced a lower driving force. The cross point met by the weight loss rate profiles of the control sample, and the water suggested the moment when the evaporation flux of the two samples was the same (Figure 6A). The concentration of melon juice corresponding to this point was 49.66%. Similar to our results, the energy efficiency, specific water removal rate, and exergy efficiency reached maximum values at about 30% total soluble solid content during the concentration of pomegranate juice. Those decreased in the further concentration process (42).

The weight loss rate between the treatments was different after deducting the effect of water evaporation (Figure 6B). The weight loss rate of the HP2-2 treatment was the highest, while that of the UHT treatment was the lowest. The HP reduced the surface tension between the aromatic components and the water matrix in the HP2-2, thus enhancing the volatilization of the aromatic components. The results further confirmed the results of the surface tension analysis.

The results of the surface tension and weight loss rate of the melon juice confirmed our prediction. Therefore, reducing the surface tension might be one of the reasons that the HP treatment enhanced the concentration of the total volatile components of melon juice.

## Conclusions

A total of 57 volatile compounds were identified from melon juice by GC-MS analysis. Among them, eight major aromatic components were identified: 2-methyl-1-butyl acetate, ethyl acetate, (E, Z)-3,6-nonadien-1-ol, (Z)-3-nonen-1-ol, acetal, nonanal, octanal, and β-ionone. β-Ionone was detected as the major aromatic component in melon juice for the first time. Its OAV value was as high as 587.87 in the HP2-2. The total volatile concentration of melon juice was highest (532.27 μg/kg) in the HP2-2 group, which was 1.54 and 3.77 times that of the control and the UHT treatment, respectively. Meanwhile, the total concentration of 8 aromatic components in the HP2-2 group was highest, which was 1.49 and 6.99 times that of the control and UHT, respectively. Hence, the HP2-2 was considered the optimal parameter of the HP treatment.

The potential mechanism of the HP treatment was explored by measuring the surface tension and the weight loss rate. The HP2-2 reduced the surface tension between aromatic components and the water matrix and enhanced the weight loss rate of the melon juice, thereby enhancing the volatilization of aromatic components. This result provided more explicit evidence for the HP flavor retention technology.

## Data availability statement

The raw data supporting the conclusions of this article will be made available by the authors, without undue reservation.

## Author contributions

CZ, XL, and YW contributed to conception and design of the study. XL, YS, and RW organized the database. XL and RW performed the statistical analysis. XL wrote the manuscript. CZ and XL reviewed the manuscript. CZ and HL supervised this study. All authors contributed to manuscript revision, and approved the submitted version.

## Funding

This research was funded by National Natural Science Foundation of China (32172237 and 82074276), China Agricultural Research System (CARS-25), and Collaborative Innovation Center of the Beijing Academy of Agricultural and Forestry Sciences (KJCX201915).

## Conflict of interest

The authors declare that the research was conducted in the absence of any commercial or financial relationships that could be construed as a potential conflict of interest.

## Publisher's note

All claims expressed in this article are solely those of the authors and do not necessarily represent those of their affiliated organizations, or those of the publisher, the editors and the reviewers. Any product that may be evaluated in this article, or claim that may be made by its manufacturer, is not guaranteed or endorsed by the publisher.
